# The Inhibition of Radial and Axial Micromovement of Bone Scaffold with Gelfoam^®^ and Titanium Mesh Fixation and Its Effects on Osteointegration

**DOI:** 10.3390/mps2010020

**Published:** 2019-02-26

**Authors:** Jane Kwon, Dong Joon Lee, Mallory Kocher, Yong-Il Kim, Te-Ju Wu, John Whitley, Ching-Chang Ko

**Affiliations:** 1 Oral and Craniofacial Health Sciences Research, School of Dentistry, University of North Carolina, CB #7455, Chapel Hill, NC 27599, USA; jakw@email.unc.edu (J.K.); dongjoon_lee@unc.edu (D.J.L.); mekocher@live.unc.edu (M.K.); orthowilliam@gmail.com (T.-J.W.); John_Whitley@unc.edu (J.W.); 2 Department of Orthodontics, School of Dentistry, Pusan National University, Yangsan 50612, Korea; kimyongil@pusan.ac.kr; 3 Department of Orthodontics, Chang Gung Memorial Hospital, Kaohsiung 83301, Taiwan; 4 Department of Orthodontics, School of Dentistry, University of North Carolina, CB #7455, Chapel Hill, NC 27599, USA

**Keywords:** critical-sized defect (CSD), Gelfoam^®^, micromovement, osteointegration, titanium mesh

## Abstract

A major drawback of nanocomposite scaffolds in bone tissue engineering is dimensional shrinkage after the fabrication process. Shrinkage yields gaps between the scaffold and host bone in the defect site and eventually causes failure in osteointegration by micromovement. The present study was conducted using titanium (Ti) mesh and Gelfoam^®^ to prevent radial and axial micromovement, respectively. A critical-sized defect (CSD) was created in the center of the calvarium of Sprague Dawley rats to implant porous polydopamine-laced hydroxyapatite collagen calcium silicate (HCCS-PDA), a novel nanocomposite scaffold. Gelfoam^®^ was applied around the edge of the defect, and then the HCCS-PDA scaffold was inserted in the defect area. Ti mesh was placed between the periosteum and skin right, above the inserted scaffold site. There were two test groups, with a fixture (Gelfoam^®^ and Ti mesh) and without a fixture, each group contained five animals. The rats were sacrificed after three months post-operation. The explanted calvaria underwent micro-CT scanning and a push-out test to quantify osteointegration and mechanical strength between the scaffold and host bone. Histological analysis of undecalcified bone was performed by grinding resin infiltrated calvaria blocks to prepare 10 μm slices. Osteointegration was higher in the group with fixation than without fixation. Movement of the HCCS-PDA scaffold in the gap resulted in diminished osteointegration. With fixation, the movement was inhibited and osteointegration became prominent. Here we present a successful method of preventing axial and radial movement of scaffolds using Gelfoam^®^ and Ti mesh. Applying this fixture, we expect that an HCCS-PDA scaffold can repair CSD more effectively.

## 1. Introduction

The tissue engineering (TE) of bone regeneration can enhance the field of plastic, reconstruction, and orthopedic surgery by repairing large bone defects rapidly and efficiently. Currently, TE bone regeneration is performed by implanting scaffolds seeded with stem cells into the defect site in order to repair with newly formed bone. In the context of scaffold materials, synthetic nanocomposites more closely mimic the structure and composition of natural bone, which can replace currently available graft options, such as autografts, allografts, and xenografts. Recent 3D printing technology further broadened the application of the nanocomposite materials on scaffold fabrication by controlling the internal environments such as pore size, porosity, and pore distribution.

This 3D porous nanocomposite scaffold is most commonly tested for its osteogenic potential in surgeries using animal models. The critical-sized defect (CSD, 8 mm) model is well defined in rats and can be utilized to investigate bone scaffolds’ osteogenic potential in vivo [1,2]. Ceramic or nanocomposite scaffolds have a major limitation: They shrink during the fabrication process. Consequently, this shrinkage creates a space between the host bone and the scaffold, allowing the scaffold to move freely and uncontrollably. This causes instability or micromovement of the scaffold during the experiment, and such movement critically hinders the ability of bone to regenerate.

Another obstacle that micromovement presents in bone regeneration using scaffolds, is the development of fibrous tissues instead of calcified tissues. In long bone fractures, the development of fibrocartilage and poor healing was reported to be due to the constant rupture of the capillaries with gross movements of the bone [3,4]. Additionally, Yamaji et al. reported that healing was either delayed or the bone formation was in non-union when the defect size increased [5]. For successful bone ingrowth, the scaffold has to have minimal movement so the tissue can migrate and merge with the scaffold and angiogenesis can proceed, which is essential for bone regeneration, as it allows cells to receive nutrients and provides protection against foreign pathogens. Then the tissue can anchor to the existing bone and promote new bone development. Without maximum stability, the scaffold disrupts all bone formation and angiogenesis, increases inflammation in the area, and ultimately inhibits healing of bone defects [6].

Yamaji et al. reported that inhibition of micromovement using fixtures during the bone healing period resulted in increased new bone formation [5]. Previously, plate-screw internal fixation and commercial titanium (Ti) mesh were used to prevent movement of the scaffold. However, the inconvenience of dealing with small screws, delay of surgery time, and the addition of another invasive procedure could cause higher rates of mortality after small animal surgery.

Our goal is to create a fixation that will prevent axial and radial micromovement of the scaffold and optimize bone regeneration. In order to prevent micromovement of the scaffold, we will be testing if the addition of a Ti mesh plus Gelfoam^®^ fixture gives support for the scaffold and, as a result, increases the amount of bone tissue growth to the host bone compared to the condition without the scaffold fixture. Typically, a layer of adhesive gel is used to prevent horizontal movement of the scaffold. On top of this, one must also place a mesh to prevent the scaffold from moving vertically. A polydopamine-laced hydroxyapatite collagen calcium silicate (HCCS-PDA) nanocomposite scaffold was inserted into a cranial defect of a rat. In order to determine if Gelfoam^®^ and mesh prevent micromovement, two groups were tested, and their percent of tissue anchored to bone was compared after 12 weeks of implantation in the rat calvarial critical-sized defect. The control group contained the scaffold without any fixture and the experimental group contained a scaffold with a fixture, Gelfoam^®^ and Ti mesh.

## 2. Materials and Methods

### 2.1. Implantation of Scaffold in CSD of Rat Calvaria

**Figure 1:**
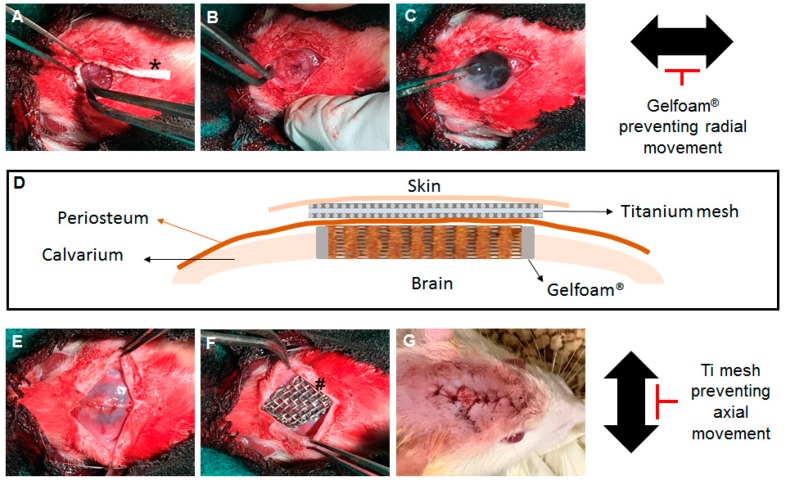
Surgical procedure of scaffold implantation into critical-sized defect on rat calvaria. Gelfoam^®^ strip was inserted into the edge of critical-sized defect of rat calvaria (*) and Titanium mesh (#) was implanted on the sutured periosteum.

Animal procedures were approved by the Animal Care and Use Committee (Animal protocol No. 18-201.0-A) and followed the guidelines for Animal Care and Use Committee (ICAUC) at the University of North Carolina Chapel Hill. Previously developed biomimetic bone scaffold material [7], the HCCS-PDA was fabricated using a printed mold method; the mold contained interconnected pores that were 300 μm in size. Two test groups (with and without Ti mesh plus Gelfoam^®^) with five Sprague Dawley rats (Charles River, Wilmington, MA, USA; about 250 to 300 g, seven weeks old) in each group were used in this experiment. The rats were anesthetized with an intraperitoneal injection of 95 mg/kg ketamine/xylazine. After shaving and sterilizing the head with 70% ethanol and iodine solution, a 2 cm midline incision was made over the calvaria, and both the periosteum and skin were retracted with hemostats to expose the calvarium. An 8 mm critical size defect was created with a trephine bur. Adequate irrigation was supplied during the procedure. The calvaria was drilled until the calvaria disk felt loose, and then any remaining connections were cut using the edge of the burr. The calvaria disk was lifted up using a probing tool and the connected tissues were cut. The Gelfoam® (2 by 10 mm) was inserted around the edge of the defect (Figure 1A,B). An HCCS-PDA scaffold was carefully inserted into the defect without interrupting the Gelfoam® (Figure 1C). After the scaffold fit in the defect tightly, the periosteum was closed using 5-0 catgut sutures (Figure 1E), Ti mesh was placed above the scaffold (Figure 1F), and the skin was sutured with 4-0 polypropylene (Figure 1G). After 8 weeks, the animals were euthanized using carbon dioxide gas, and the calvaria was retrieved after fixation of the implanted sites in 10% formalin for 7 days at 4 °C. The explanted calvaria was then stored in 70% ethanol at 4 °C for further analysis.

### 2.2. Micro-CT Analysis

Fixed calvarial explants were scanned using micro-CT (µCT 40; Scanco Medical, Brüttisellen, Switzerland) under the following conditions: 70 kV, 114 mA, and 200 ms integration time. After 3D reconstruction using CTAn (Skyscan Company, Aartselaar, Belgium), the percentage of osteointegration between host bone and scaffold around the edge of the defect was measured using Image J software (U.S. National Institutes of Health, Bethesda, MD, USA). The percentage of osteointegration between host bone and scaffold was calculated by dividing the length of the osteointegrated area in the CSD by the total circumference of the CSD. The data were presented as the average ± standard deviation.

### 2.3. Mechanical Analysis

Using a custom-made test apparatus, the push-out strengths of the calvarial implants were tested. The apparatus was designed using the specifications of Spicer et al. [2]. After mounting the test apparatus onto a mechanical testing instrument (Instron ElectroPuls E3000, Norwood, MA, USA), a 6 mm probe, which was attached to a 250 N load cell, was aligned concentrically with a 10 mm diameter hole in the base. Following removal from cold PBS, the tissue samples were placed so that the implant was concentric with the hole, with the cerebral surface facing the probe. The samples were clamped on all four sides to maintain the position while testing. The probe applied a vertical force on the implant at a rate of 0.5 mm/min until a peak force was reached.

### 2.4. Undecalcified Section Preparation for Histological Analysis

The explanted calvaria was dehydrated and subsequently infiltrated with resin (Technovit, Heraeus Kulzer GmbH, Hanau, Germany) for 32 days. The gross tissue was adhered to a mold with Technovit 7230 and embedded with Technovit 7200 embedding media. Air bubbles in the mold were eliminated in a vacuum chamber. The sample was polymerized for 2 to 6 h with yellow and blue lamps. The slides were mounted to both sides of the sample, and the sample was cut to the area of interest (EXAKT Model 310 sectioning saw, Norderstedt, Germany). The desired sections were ground until their thickness reached between 65 and 70 mm (EXAKT Model 400 microgrinding system, Norderstedt, Germany). Once polished, sections were stained using Stevenel’s blue solution and counterstained with Van Gieson’s stain. Color images of the stained sections were acquired with a Nikon inverted microscope apparatus with a Nikon Eclipse Ti-U digital camera (Nikon Instruments Inc., Melville, NY, USA).

### 2.5. Statistics

For statistical analysis, a Student’s *t*-test was used to compare the means between groups with and without fixation and data are presented as the mean ± standard deviation.

## 3. Discussion

The shrinkage of the ceramic scaffolds and irregularities in the defect hinders a tight fit in the CSD. Moreover, the brittle characteristic does not allow the scaffold to be cut or trimmed, which would damage the scaffold structure. These limitations usually cause the gap between the scaffold and host calvarial bone to allow micromovement or dislodgement of the scaffold in the CSD. In previous studies, wrong conclusions may have been postulated for the osteogenic effect of ceramic scaffolds due to movement between the bone and the scaffold.

Many studies in bone grafting procedures and long bone defect repairs have shown improvements in bone regeneration while using a fixture to control micromovement [8,9,10,11]. The particular fixture mechanism in our study not only helped to secure the scaffold, but also improved osteointegration by effectively anchoring the new bone to the host bone.

Gelfoam^®^ is a gelatin sponge prepared from purified pork skin gelatin. Gelfoam^®^ has been tested as a graft material. Finn et al. evaluated Gelfoam^®^ with four different hemostatic agents for their regenerative potential of bone in an iliac crest defect in a canine model [12]. They found new bone and hematopoietic tissue using Gelfoam^®^. After two-month implantation in our study, Gelfoam^®^ was incorporated into the new bone, without any significant foreign-body reaction. The main benefit of Gelfoam^®^ was its ability to prevent radial movement. However, additional benefits were discovered throughout the experiment. Gelfoam^®^ consists of type 1 collagen, which makes up the majority of the organic phase of natural bone. As a flexible material, Gelfoam^®^ effectively occupied space created between the scaffold and the CSD as a result of ceramic scaffold shrinkage, and facilitated osteointegration and tissue migration at the interface between the native bone and scaffold by preventing micromovement. In addition, Gelfoam^®^ inhibited bleeding during the surgical process by inducing rapid coagulation and expedited the healing process of the bone defect.

Paired with Gelfoam^®^, the Ti Mesh inhibited axial movement that resulted from the dislocation of the shrunk scaffold from the CSD. In addition, the Ti mesh used in this experiment was biocompatible and could easily be customized to form the shape of the defected area. Therefore, the combination of Ti Mesh and Gelfoam^®^ minimized axial and radial movement of the scaffold, and thus, inhibited micromovement. While the individual materials might not have been sufficient in preventing micromovement or dislodgement, the combination of the two materials has proven to be effective in fixing the scaffold in the CSD.

A common setback in the experiment is the damaging of the scaffold when implanting it into the defected area. In several rats, the scaffold broke inside the CSD. The breakage caused an increase in open space in the defect site. Such space and increase in scaffold pieces resulted in aggravation of random micromovement, and ultimately, in the diminution of osteointegration by preventing anchorage of new tissue onto the bone.

Micro-CT visually eliminated any fibrous tissue and imaged only the integration of bone at the host bone-scaffold interface. The micro-CT results showed a significant increase in osteointegration in the group with fixtures compared to the group without, thereby confirming the effectiveness of the fixation to minimize the micromovement of the scaffold.

The outcome of the push-out test indicated that the group with the fixture was significantly more resistant against the push-out force, which implied that the fixture increased osteointegration between the scaffold and host bone.

The conventional decalcified histology removes calcium from samples by preserving only soft tissue, mostly collagen, which complicates distinguishing newly regenerated mineralized bone in the samples. Therefore, undecalcified histology that maintains calcium composition in the bone tissue of the samples was utilized in the experiment. In addition to micro-CT data, which showed the entire structure of rat calvaria, detailed histological images of sample cross-sections corroborated the effectiveness of fixtures in enhancing osteointegration.

Additional testing, such as the use of immunohistochemistry antibody staining, could be used to further corroborate the amount of new bone if the results of the current tests were not as significant.

Our results demonstrate that the Ti mesh plus Gelfoam^®^ fixation increased the percent of osteointegration between the host bone and scaffold by preventing movement of the scaffold. One can infer that using this fixture minimized micromovement and enhanced the healing process of bone regeneration. Our fixture mechanism, that utilizes Ti mesh plus Gelfoam^®^ fixation, should improve numerous therapeutic applications. With the enhanced osteointegration, innovative techniques, such as cell seeding and scaffold optimization, can be incorporated to effectively treat larger bone defects in the future.

## 4. Results

**Figure 2:**
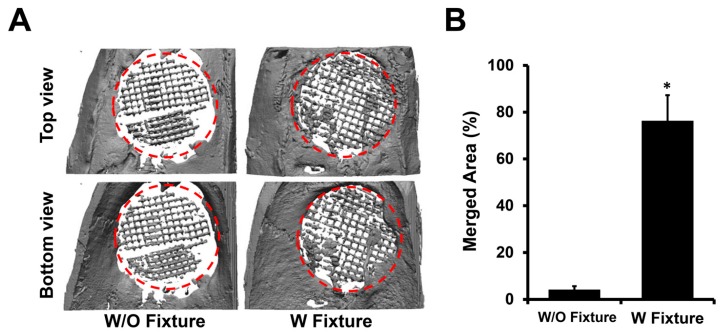
(**A**) Micro-CT image of calvarial defects after 12 weeks of scaffold implantation with and without Gelfoam^®^ and Ti mesh. (**B**) Merged area between host bone and scaffold in the defect site was quantified using Image J software (*n* = 5, * *p* <0.05).

After three months post-implantation, rats were sacrificed and the calvaria was excised for micro-CT, push-out, and histological analysis. Micro-CT allowed 3D reconstruction of calvaria (Figure 2A). From the reconstructed image, osteointegrated areas between host bone and scaffolds were calculated to be 76.34 ± 10.92% and 4.18 ± 1.47% for the groups with and without a fixture, respectively (Figure 2B).

Although figures of an empty defect were not shown, an empty CSD did not heal after three months, confirming the defect to be a true CSD. As indicated by the higher percentage, the group with a fixture demonstrated significantly improved osteointegration between the host bone and scaffold. The elimination of micromovement is crucial in inducing osteointegration when using a bone growth scaffold.

**Figure 3:**
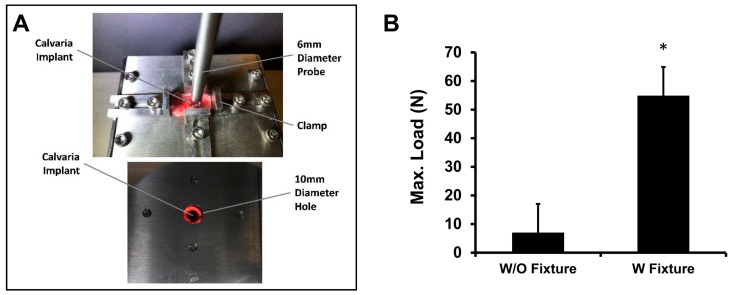
(**A**) Apparatus of push-out test, (**B**) mechanical strengths of scaffold after 12 weeks of implantation with and without Gelfoam^®^ and Ti mesh. A rat calvarial explant was placed on the apparatus and fixed by pairs of clamps as increasing amount of load was applied on the scaffold.

The mechanical strength that results from osteointegration was measured using a push-out test, distinguishing bone-scaffold interface conditions as healing progressed (Figure 3A). The failure load and stiffness of the bone-scaffold interface with fixtures (54.9 ± 32.21 N) was significantly higher than that without a fixture (7.02 ± 2.89 N) measured after a 12-week healing time (Figure 3B).

Histological evaluation of the osteointegration was assessed in the region between host bone and scaffold from each group without a fixture and with a fixture. The undecalcified resin sections were stained with Stevenel’s Blue and Van Gieson’s stain to identify whether newly formed bone (red color) merged with the scaffold at the edge of the defect site. The undecalcified histology demonstrated that the scaffolds in the group with the fixture merged with the host bone, while the group without the fixture failed to induce osteointegration, as indicated by the absence of staining at the interface of the native bone and scaffold.

**Figure 4:**
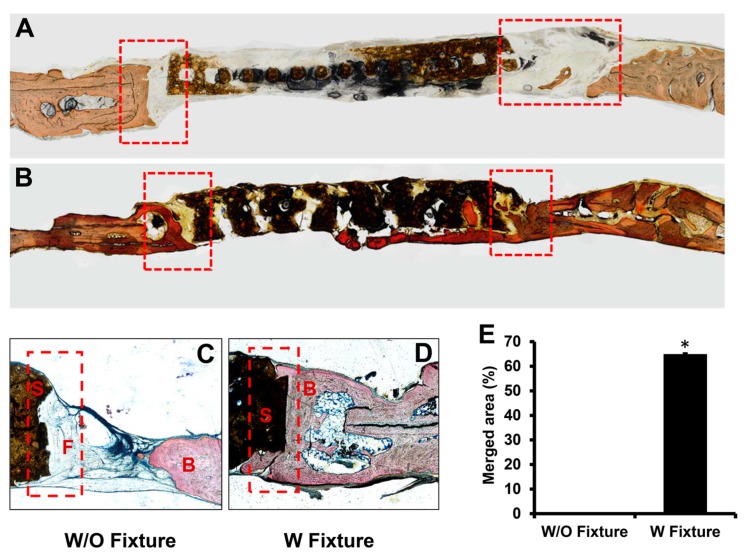
Histological section of the area between host bone and scaffold after 12 weeks of implantation without (**A**,**C**) and with (**B**,**D**) fixture. Sections were stained with Stevenel’s Blue and Van Gieson’s stain after grinding process. Dotted rectangles represented region of interest (ROI). S: scaffold F: fibrous tissue and B: bone. The merged area between host bone and scaffold was quantified in percentage using Image J software, (*n* = 5, * *p* <0.05) (**E**).

In the group without a fixture, osteointegration was rarely observed at the periphery of the defect and intervening tissue was mainly fibrous connective tissue (Figure 4A,C). A successful osteointegration was observed at the interface between the host bone and scaffold for the group with a fixture (Figure 4B,D). Quantitative analysis of osteointegration between the host bone and scaffold demonstrated 64.79 ± 0.45% and 0% integrations for the groups with and without a fixture, respectively (Figure 4E).

## References

[1] Schmitz J.P., Hollinger J.O. (1986). The critical size defect as an experimental model for craniomandibulofacial nonunions. Clin. Orthop. Relat. Res..

[2] Spicer P.P., Kretlow J.D., Young S., Jansen J.A., Kasper F.K., Mikos A.G. (2012). Evaluation of bone regeneration using the rat critical size calvarial defect. Nat. Protoc..

[3] Rhinelander F.W. (1974). Tibial blood supply in relation to fracture healing. Clin. Orthop. Relat. Res..

[4] Claes L., Eckert-Hubner K., Augat P. (2002). The effect of mechanical stability on local vascularization and tissue differentiation in callus healing. J. Orthop. Res..

[5] Yamaji T., Ando K., Wolf S., Augat P., Claes L. (2001). The effect of micromovement on callus formation. J. Orthop. Sci..

[6] Loi F., Córdova L.A., Pajarinen J., Lin T., Yao Z., Goodman S.B. (2016). Inflammation, fracture and bone repair. Bone.

[7] Lee D.J., Lee Y.-T., Zou R., Daniel R., Ko C.-C. (2017). Polydopamine-Laced Biomimetic Material Stimulation of Bone Marrow Derived Mesenchymal Stem Cells to Promote Osteogenic Effects. Sci. Rep..

[8] Lee D.J., Diachina S., Lee Y.T., Zhao L., Zou R., Tang N., Han H., Chen X., Ko C.-C. (2016). Decellularized bone matrix grafts for calvaria regeneration. J. Tissue Eng..

[9] Perren S.M., Regazzoni P., Fernandez A.A.D. (2014). Biomechanical and biological aspects of defect treatment in fractures using helical plates. Acta Chir. Orthop. Traumatol. Cech..

[10] Goodman S.B. (1994). The effects of micromotion and particulate materials on tissue differentiation. Bone chamber studies in rabbits. Acta Orthop. Scand. Suppl..

[11] Gao S.-S., Zhang Y.-R., Zhu Z.-L., Yu H.-Y. (2012). Micromotions and combined damages at the dental implant/bone interface. Int. J. Oral Sci..

[12] Finn M.D., Schow S.R., Schneiderman E.D. (1992). Osseous regeneration in the presence of four common hemostatic agents. J. Oral Maxillofac. Surg..

